# Type I and III Interferon in the Gut: Tight Balance between Host Protection and Immunopathology

**DOI:** 10.3389/fimmu.2017.00258

**Published:** 2017-03-14

**Authors:** Johanna Pott, Silvia Stockinger

**Affiliations:** ^1^Sir William Dunn School of Pathology, University of Oxford, Oxford, UK; ^2^Institute of Animal Breeding and Genetics, University of Veterinary Medicine, Vienna, Austria

**Keywords:** interferon, intestinal mucosa, colitis, enteropathogens, IFN-λ, type 1 IFN, inflammatory bowel diseases, coeliac disease

## Abstract

The intestinal mucosa forms an active interface to the outside word, facilitating nutrient and water uptake and at the same time acts as a barrier toward the highly colonized intestinal lumen. A tight balance of the mucosal immune system is essential to tolerate harmless antigens derived from food or commensals and to effectively defend against potentially dangerous pathogens. Interferons (IFN) provide a first line of host defense when cells detect an invading organism. Whereas type I IFN were discovered almost 60 years ago, type III IFN were only identified in the early 2000s. It was initially thought that type I IFN and type III IFN performed largely redundant functions. However, it is becoming increasingly clear that type III IFN exert distinct and non-redundant functions compared to type I IFN, especially in mucosal tissues. Here, we review recent progress made in unraveling the role of type I/III IFN in intestinal mucosal tissue in the steady state, in response to mucosal pathogens and during inflammation.

## Introduction

The intestinal tract is a major entry site for viruses and bacteria. Mucosal innate and adaptive immune cells are equipped to respond to and fight invading pathogens. At the same time, the intestinal lumen is densely colonized by commensal microflora, which at steady state does not provoke an exacerbated inflammatory response. The intestinal lumen is separated from the underlying sterile lamina propria harboring the body’s largest immune cell compartment by a single layer of polarized intestinal epithelial cells (IECs). This epithelial cell layer undergoes rapid and perpetual self-renewal without disrupting the functional integrity of cell–cell junctions. In addition, IECs not only form a passive physical barrier but also participate actively in the immune response against major enteric pathogens and cross talk with the commensal flora (1, 2). However, pathogenic viruses, bacteria, and parasites exploit opportunities for breaching the epithelial barrier.

Upon infection, host cells communicate by means of production and secretion of signaling molecules. Interferons (IFN) are a large family of cytokines with diverse functions during a successful host defense. The family of type I IFN comprises more than 20 members with multiple IFN-α and one IFN-β being the most important. Classically, the most prominent function of type I IFN is to induce antiviral immunity, whereas IFN-γ, the only type II IFN, promotes the response to intracellular bacteria. However, a vast amount of studies has found that type I IFN are also produced during bacterial infection. In contrast to their action in viral infections, their activity against bacteria can be either favorable or detrimental for the host (3–6).

Recently, a novel family of IFN, the type III IFN or IFN-λ family, was described (7, 8). This family consists of IFN-λ1, IFN-λ2, IFN-λ3 (also called IL-29, IL-28A, and IL-28B), and IFN-λ4 in humans, whereas mice have only two functional genes encoding IFN-λ (*Ifnl2* and *Ifnl3*) and two *Ifnl1* pseudogenes (9). Similar to type I IFN, type III IFN are induced by viral infection and show antiviral activity. However, they are structurally distinct from type I IFN and interact with a heterodimeric class II cytokine receptor consisting of the IFN-λR1 (also called IL-28Rα) chain in complex with the IL-10R2 chain, opposed to the type I IFN receptor (IFNAR).

A number of studies have addressed the functional importance of type III IFN compared to type I IFN in the context of viral infections (10–15). Less is known about the role of type I IFN and almost nothing on the role of type III IFN in the host defense against bacterial enteropathogens, intestinal homeostasis, and colitis. Therefore, we review recent progress made on the importance of type I and III IFN during enteric viral infections and focus on the role of type I IFN in the intestinal mucosal tissue during steady state, in response to bacterial infections and during inflammation.

## Induction of Type I and III IFN

The induction of type I and III IFN has been recently reviewed elsewhere (16), therefore we will only briefly summarize the major mechanism leading to IFN expression. Virtually all cells are equipped with the machinery to recognize viral infection and express type I and III IFN in response. Similar stimuli and pathways lead to the expression of type I and III IFN; however, differences between cell types as well as in magnitude and kinetics have been described (14, 16, 17). Comparable expression patterns of type I and III IFN result from a similar requirement of transcription factors for the expression of their encoding genes, such as IFN regulatory factors (IRFs) and NF-κB. There are however some differences in the promoter region, with IFN-β expression relying on the binding of the constitutively expressed IRF-3 to its promoter, which allows rapid induction. By contrast, IFN-α requires IRF-7 binding, which is an interferon-stimulated gene (ISG) itself and needs to be upregulated in most cell types following infection (3). Type III IFN are more dependent on the activation of NF-κB (18) and require the combined action of IRFs and NF-κB for full induction (19–21).

During systemic viral infections hematopoietic cells are the major source of type I IFN. Plasmacytoid dendritic cells (pDCs), which are designated as being the “professional” type I IFN-producing cells, produce large amounts in response to a wide range of viruses, parasites, and bacteria and are particularly important in the early phase of type I IFN production (22–24). However, depending on the infectious agent, myeloid cells are also involved in systemic type I IFN production. During systemic *Listeria* infection, the vast amount of systemic IFN-β production is independent of pDCs but seems to be produced by LysM-Cre-expressing macrophage/monocyte-like cells including TipDCs but not neutrophils (25–27). In the intestinal lamina propria, dendritic cells (DCs) as well as mononuclear phagocytes produce IFN-β and IFN-α5 in the steady state (14, 23, 28).

Epithelial cells are thought to be the major producer of type III IFN at steady state and during enteric viral infection, while lamina propria leukocytes (LPLs) also produce type III IFN under certain conditions (14, 29). Intraepithelial lymphocytes produce IFN-α and IFN-λ upon TCR activation, which contributes to protection during norovirus infection (30). Moreover, Th17 cells are the main source of IFN-λ in psoriatic lesions of the skin (31).

Bacteria trigger similar intracellular signaling cascades to viral infections and many bacterial infections lead to the production of type I IFN [reviewed in Ref. (32, 33)]. Induction of type III IFN has been demonstrated only for a limited number of bacterial species. A human epithelial colon cancer cell line expresses type III IFN upon infection with Gram-positive bacteria such as *Listeria monocytogenes* (34, 35), *Staphylococcus aureus*, and *Enterococcus faecalis* but fails to produce considerable amounts of type III IFN when infected with Gram-negative bacteria such as *Salmonella enterica* ssp. Typhimurium, *Shigella flexneri*, and *Chlamydia trachomatis* (35). Induction seems to be cell type, species, and gene specific (36, 37).

## Signaling in Response to IFN

Binding of IFN to their corresponding receptors triggers the stimulation of a Janus kinase (JAK)–signal transducer of transcription (STAT) pathway. The type I IFN receptor (IFNAR) consists of two subunits, IFNAR1 and IFNAR2. Engagement of IFNAR with its ligand ultimately results in the activation of the transcription factor complex ISGF3 comprised of STAT1/STAT2 heterodimers in conjunction with IRF-9 and subsequently the induction of ISGs (3, 38).

The type III IFN receptor consists of the unique IFN-λR1 chain and the IL-10R2 chain, which is shared with the IL-10 receptor. Engagement of this receptor complex results in the activation of a signal transduction cascade in a manner highly similar to that caused by type I IFN signaling. Interestingly, signaling by type III IFN is additionally regulated at the level of receptor expression. Whereas IFNAR is ubiquitously present, the IFN-λR1 chain of the type III IFN receptor is only expressed in a limited number of cell types, preferentially located at mucosal surfaces. Epithelial cells in mucosal tissues are a major target of type III IFN (39, 40). Additional responsiveness to type III IFN has recently been suggested for a restricted panel of immune cells (9). Type III IFN was proposed to have a role in the direct regulation of NK cell effector function (41). A suppressive function of type III IFN in autoimmune and inflammatory diseases was also proposed recently. In a model of collagen-induced arthritis, treatment with type III IFN inhibits the recruitment of IL-1β-expressing neutrophils, which have been shown to express high levels of IFN-λR1 and respond directly to type III IFN (42). In addition, there are controversial data on the responsiveness of T cells, DCs, and monocytes to type III IFN (9). In human cells, expression of the type III IFN receptor seems to be less restricted than in mouse cells and a wider panel of immune cells, including B cells, is responsive to type III IFN (43).

Signaling by IFNs induces the transcription of hundreds of ISGs. These include pattern-recognition receptors, antiviral effectors such as myxovirus resistance (Mx) gene 1 and 2, pro-apoptotic genes, MHC class I genes, inducible nitric oxide synthase, and genes encoding members of the GTPase superfamily which alter the maturation of phagosomes to counteract pathogen strategies based on survival in intracellular compartments. Moreover genes involved in the desensitization to IFNs are also induced, allowing cells to recover from the IFN response (38, 44). The importance of IFNs in the immediate defense against pathogens has been shown by the generation of gene-targeted mice. Mice deficient in components of the type I IFN signal transduction pathway are highly susceptible to a variety of viruses (5, 45). The role of type I IFN in bacterial infections is more complex. Whereas type I IFN protect mice against systemic infection with most extracellular bacteria tested, they exacerbate disease during infection of mice with *L. monocytogenes* or *Mycobacterium tuberculosis* (3–6).

## Enteric Viral Infections and IFN

Studies investigating the functional importance of type I IFN versus type III IFN in the context of systemic viral infections found a dominant phenotype for type I IFN and only a small contribution of type III IFN in the absence of type I IFN. The first indication for a tissue-specific role of type III IFN arose from studies with organ-tropic viral infections suggesting that type III IFN are important in enforcing and strengthening the antiviral response at mucosal sites (Table 1) (10–12, 46–48). The gastrointestinal tract, lung, vagina, and salivary glands respond strongly to systemic IFN-λ expression (40). In the lung and gastrointestinal tract, epithelial cells were identified to express high levels of the type III IFN receptor and represent the major target of type III IFN (11). These findings explain why mice deficient for both IFN systems are more susceptible to lung-tropic viruses, such as influenza A and B virus, respiratory syncytial virus, and severe acute respiratory syndrome coronavirus than single type I IFN receptor-deficient mice (11). The remaining part of this section focuses on the role of type III IFN in enteric viral infections.

**Table 1:** **Role of type I interferons (IFN) and type III IFN during enteric viral infections**.

	Role of type I IFN	Type III IFN
Rotavirus	Type I IFN protect from systemic infection and heterologous oral infection (49, 50)	Type III IFN protect from oral homologous infection by restriction of replication within intestinal epithelial cells (IECs) (12, 29)
Norovirus	Type I IFN protect from systemic spread of acute norovirus infection (48, 51)	Type III IFN protect from persistent norovirus infection; treatment with IFN-λ clears persistent infection (15)
Reovirus	Type I IFN restricts reovirus replication in lamina propria leukocyte (14)	Type III IFN restricts reovirus replication in IECs and fecal shedding (14)
EMCV	IFN-α treatment reduces titer in hearts during systemic infection (47)	Type III IFN does not protect during systemic infection (47); type III IFN protects from oral infection (52)

### Rotavirus

Rotavirus belongs to the family of reoviridae and infection of humans leads to severe diarrhea in children younger than 5 years. The susceptibility of infants can be recapitulated in a mouse model, where suckling mice are highly susceptible to infection compared to adult mice. The strict host cell tropism of rotavirus for IECs makes it a clean model to study epithelial-specific effects of IFN.

Mice can be infected with a homologous strain of murine rotavirus or with a heterologous strain such as rhesus or simian rotavirus. Homologous strains are better equipped to evade the host immune response, which generally leads to higher viral titers and a more severe pathology at a lower infectious dose (49).

A protective role of type I IFN and IFN-γ has been questioned, since mice impaired in type I IFN or IFN-γ signaling infected with a murine rotavirus strain do not show differences in viral load, and treatment with either type I IFN or IFN-γ did not result in a clinical benefit (53). However, simian and rhesus rotavirus show enhanced systemic replication in mice deficient for type I IFN and IFN-γ receptor or STAT1.

By contrast, type III IFN were protective in a homologous infection model of suckling and adult mice (12). Of note, a very distinct cell tropism for type III IFN responsiveness in the intestine was reported: IECs were solely activated by type III IFN and are not responsive to type I IFN, whereas cells in the lamina propria respond to type I IFN induced during viral infection (12). Supporting these findings it was shown that IL-22 augments the antiviral effects of type III IFN signaling and contributes to the protective effect during homologous rotavirus infection (29). However, this model has been questioned by another study reporting type I IFN- and type III IFN-mediated protection only for heterologous but not for homologous rotavirus infection of suckling mice (50). Experimental discrepancies between those studies are not apparent suggesting that flora differences between mouse facilities or genetic strategy of the knock-out mouse lines might impact on the efficacy of IFN signaling. Of note, Lin et al. reported age-dependent responsiveness of IECs toward IFNs with neonatal IECs being responsive to both type I IFN and type III IFN, whereas adult IECs were responsive to type III IFN only (50).

### Norovirus

Norovirus is the cause of the majority of non-bacterial gastroenteritis in adults. In contrast to rotavirus, the host cell tropism of norovirus is broad and not fully characterized. *Ex vivo* and most *in vivo* studies could not show productive virus replication in IECs (54). Phagocytes allow productive virus replication and during *in vivo* infection, virus was detected in LPLs (54, 55). Although the virus does not replicate in IECs, it has been suggested that it translocates across the epithelium or enters the host *via* M cells (56).

Type I IFN and IFN-γ restrict murine norovirus replication in macrophages and DCs *in vitro* (57, 58). *In vivo*, the antiviral activity of type I IFN mediates some protection from systemic replication of an acute strain (51) and after high-dose oral infection (59). However, local replication in the colon and fecal shedding of a persistent norovirus strain is controlled by type III IFN. Treatment with type III IFN resolves persistent infection, independent of adaptive immune responses, by acting on non-hematopoietic cells (15). By contrast, type I IFN controls the systemic spread and persistency of the acute norovirus strain CW3 by activation of the host DCs (48). These findings demonstrate the distinct cell-type specificities of type I IFN and type III IFN during infection: local protection in the colon through type III IFN stimulation of epithelial cells and prevention of systemic spread and persistency by type I IFN in myeloid cells.

The commensal bacterial flora was reported to promote norovirus persistency in the intestine and antibiotic treatment of mice prevented persistent infection with norovirus. The protective effect was only observed in the presence of functional type III IFN signaling (13). The antibiotic treatment did not alter type III IFN signaling and therefore the authors concluded that the microflora might render the virus susceptible to the antiviral action of type III IFN. Alternatively, the absence of type III IFN signaling might increase the host’s vulnerability to persistent viral infection so dramatically that minor changes by antibiotic treatment do not impact on the overall susceptibility under those conditions.

### Reovirus

**Figure 1:**
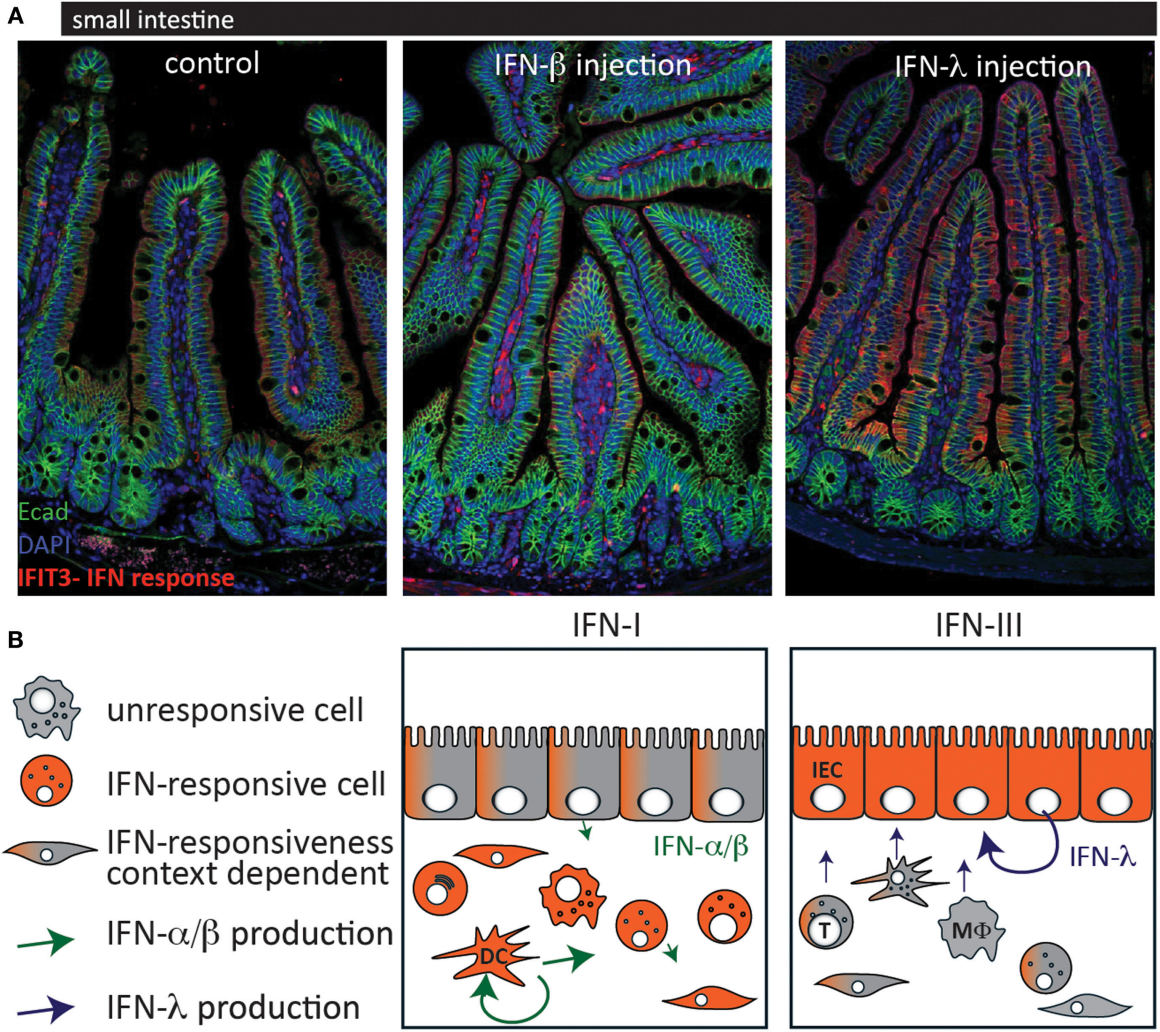
**Cell-type-specific responsiveness to type I interferons (IFN) and type III IFN at the intestinal mucosa**. **(A)** C57BL/6 mice were injected with IFN-β (1,000 U) (middle panel) or IFN-λ (1 μg) (right panel) and 3 h later, the small intestine was processed for histological assessment. Staining was performed for the interferon-stimulated gene IFIT3 (red) as a marker for IFN response and for the epithelial cell marker E-cadherin (green). **(B)** Schematic of the IFN production and responsiveness at the intestinal mucosa. When type I IFN levels are high, lamina propria cells readily respond with a strong IFN response whereas IECs are rather unresponsive but might respond under certain conditions [**(A)** middle panel; **(B)** left panel]. In contrast, IECs are the most responsive cells to type III IFN **(A,B)** right panel. Most virus-infected cells express type I IFN. Hematopoietic cells, such as plasmacytoid dendritic cells and macrophages produce the highest amounts of type I IFN whereas IECs seem to express preferentially type III IFN. T, T cell; DC, dendritic cell; MΦ, macrophage; IEC, intestinal epithelial cell.

Reovirus has a broad host cell tropism and replicates in epithelial cells and immune cells of the intestinal mucosa. After oral infection, it enters the host *via* M cells into Peyer’s patches and can spread further during infection. Type I IFN produced by hematopoietic cells is essential to limit systemic spread of the virus and to prevent lethality (60). In a study using type I IFN or type III IFN signaling-deficient mice, it was demonstrated that type III IFN signaling specifically prevents replication of the virus in IECs, whereas type I IFN signaling limits replication in lamina propria cells and systemic spread of the virus (14). This study confirms the compartmentalized action of IFN in the intestinal mucosa and provides an explanation by showing that IECs only express low levels of IFNAR (Figure 1). Furthermore, it was shown that the production of IFN is cell type specific in that IECs produce higher levels of type III IFN and LPLs predominantly produce type I IFN.

Taken together, the studies of enteric viral models with rotavirus, reovirus, and norovirus show a strong and specific responsiveness of IECs to type III IFN (12, 15, 29). Therefore, type III IFN might specifically enforce the intestinal barrier against enteric viruses and also against viral entry *via* the intestinal route. Additionally, a strong IFN response by type III IFN signaling within the epithelial lining prevents viral spreading (12, 14, 15). Studies showing that type III IFN treatment protects against oral EMCV (52) infection but not from systemic infection (47) support the conclusion that type III IFN protects the host not only from enteric viruses but also from viral entry *via* the oral route. By contrast, the contribution of type I IFN to the epithelial antiviral response in the intestine is less clear and conflicting results suggest it to be context dependent (12, 29, 50).

## Bacterial Infections

In contrast to viral infections where type I IFN and/or type III IFN usually provide an efficient host defense by triggering the production of antiviral genes, the role of type I/III IFN in the antibacterial response depends on the pathogen and the route of infection (3, 4, 61). Type I IFN signaling protects against most extracellular bacteria tested but is detrimental in the course of infection with a range of intracellular bacteria [Table 2; reviewed in Ref. (61)].

**Table 2:** **Role of type I interferons (IFN) in intestinal inflammation and bacterial infections**.

Mouse strain	Phenotype—type I IFN	Reference
**BACTERIAL INFECTION**		
***Listeria monocytogenes***		
IFNAR1^−/−-^, IFN-β^−/−^, IRF-3^−/−^, IRF-7^−/−^	Type I IFN signaling is detrimental during systemic infection	(25, 62–64)

IFNAR1^−/−^	Kernbauer et al. showed that type I IFN signaling during oral infection with the potent type I IFN inducing LO28 strain is beneficial for the host. By contrast, Pitts et al. did not observe any role for type I IFN during oral infection with the EGDe strain	(65, 66)

LysM-Cre IFNAR^fl/fl^	Lack of IFN signaling in LysM^+^ cells confers protection during systemic infection most pronounced in early infectious stages	(25)

***Salmonella Typhimurium***		
IFN-β^−/−^	Lack of IFN-β mediates increased resistance to lethality during oral *S*. Typhimurium infection	(67)

IFNAR^−/−^	IFNAR deficiency leads to increased resistance to oral *S*. Typhimurium characterized by decreased bacterial spread and weight loss but similar intestinal pathology. In accordance, type I IFN induction due to influenza coinfection exacerbates the disease and CFU but decreased intestinal immunopathology	(68)

USP18^−/−^	During *Salmonella* infection, *Usp18*-mutant mice are more susceptible to systemic (i.e., typhoid) *S*. Typhimurium infection. By contrast, in the streptomycin-induced model of typhlocolitis, mutant *Usp18* mice display lower pathology scores, low IFN-γ production but upregulated type I IFN signaling compared to control mice, resulting in earlier systemic dissemination of the bacteria and decreased survival	(69)

***Yersinia enterocolitica***		
TRIF^−/−^	IFN-β treatment protects TRIF^−/−^ mice from *Y. enterocolitica* lethality	(70)
**COLITIS MODELS**		
***T cell transfer colitis***		
IFN-α treatment	Ameliorates T cell transfer colitis	(71)

IFNAR^−/−^host	IFNAR deficiency in the host cells exacerbates colitis; indirect effect on maintenance of Foxp3^+^ Tregs	(23)

IFNAR^−/−^ T cells	Induction of colitis by IFNAR^−/−^ T cells similar to wt T cells, however, boosting type I IFN by poly(I:C) treatment attenuates T cell transfer colitis in a T cell-(IFNAR-)dependent manner	(23, 72)

IFNAR^−/−^ Tregs	Conflicting findings on the role of IFNAR signaling in Tregs for protection from T cell transfer colitis	(23, 71)

***Dextran sodium sulfate (DSS) colitis***		
CpG ODN treatment	CpG ODN protects against DSS colitis in an IFNAR-dependent manner; by contrast, La-IFN-β treatment exacerbates colitis	(73, 74)
IFN-β-expressing *Lactobacillus* (La-IFN-β)		

IFNAR1^−/−^	Type I IFN signaling suppress acute DSS colitis but delays the resolution	(73, 75)

Villin-Cre IFNAR1^fl/fl^	IFNAR deficiency in intestinal epithelial cells results in similar susceptibility to DSS colitis as wt; increased tumor burden in DSS + azoxymethane model (due to microbiota alterations)	(76)

IL-28Rα^−/−^	Increased susceptibility in IL-28Rα^−/−^, same as IL-28Rα^−/−^ IFNAR1^−/−^ DKO indicating dominant role of type III IFN	(77)

### *Listeria monocytogenes* 

Most of the pioneering studies unraveling the potential detrimental action of type I IFN in the antibacterial response have been performed using systemic infection models with *L. monocytogenes* [reviewed in Ref. (4)]. More recent studies, however, have proposed that the impact of type I IFN on the outcome of disease depends on the route of infection (65).

Mice deficient in IFNAR1, IFN-β, IRF-3, or IRF-7 are more resistant to systemic infections with *L. monocytogenes* than wild-type mice (25, 62, 64, 78). A number of mechanistic explanations for this phenotype exist, including increased death of crucial effector cells such as macrophages and T cells in response to type I IFN signaling (64, 78, 79), upregulation of IL-10 in an type I IFN-dependent manner limiting protective inflammatory responses (80, 81), and a negative cross talk between type I IFN and IFN-γ signaling (82).

In contrast to the clear detrimental effects of type I IFN in systemic *L. monocytogenes* infections, the role of type I IFN in oral *L. monocytogenes* infection has only been addressed by a limited number of studies mainly due to the lack of a suitable mouse model. For the uptake into IECs, specific interaction between the *Listeria* virulence factor internalinA (InlA) and E-cadherin expressed on IECs is required. InlA recognizes E-cadherin from human but fails to bind the corresponding domain of murine E-cadherin (83). A knock-in mouse ubiquitously expressing “humanized” E-cadherin provides a model for oral *Listeria* challenge (84). Infected germ-free colonies of this mouse line develop systemic listerosis, which can be dampened by administration of *Lactobacilli* (85). *Lactobacilli* treatment downregulates immune gene expression and in particular ISGs, which are among the most highly induced genes after *L. monocytogenes* infection (85).

Kernbauer et al. used a “murinized” *L. monocytogenes* strain LO28 expressing altered InlA recognizing mouse E-cadherin to show that, in sharp contrast to systemic infection, type I IFN signaling in response to both high-dose and low-dose intragastric *L. monocytogenes* infection is beneficial for the host. Diminished restriction of bacterial growth in the absence of type I IFN signaling resulted in exacerbated hepatic inflammation and damage (65). Different results were obtained by a more recent study using an EGDe derivative strain expressing “murinized” InlA (66). Contrasting systemic infection, which leads to strong type I IFN secretion, oral infection with this strain did not trigger robust type I IFN induction in splenocytes even when comparable bacterial burdens were present in the spleen. Neither major T cell depletion nor increased splenic IL-10 production was observed in this model and the detected infection-induced downregulation of the IFN-γ receptor on DCs and macrophages was not dependent on type I IFN signaling. Consequently, no major differences between wild-type and IFNAR1-deficient mice orally infected with *L. monocytogenes* where detected in this study (66). The discrepancies between the Kernbauer and the Pitts study might be explained by different application methods (intragastric infection compared to natural feeding) or by the use of different strains (type I IFN hyper-inducing LO28 versus EGDe). In addition, differences in the microbiota due to the housing conditions might influence the outcome of infection.

### *Salmonella enterica* Typhimurium

*Salmonella enterica* serovar Typhimurium causes gastroenteritis and is one of the most frequent causes of bacterial foodborne disease in Western countries, representing a major economic problem (86).

Oral infection of laboratory mice with *S*. Typhimurium causes typhoid-like symptoms without clinical signs of gastroenteritis and can be used to study genetic determinants of systemic infections. IFN-β^−/−^ mice show increased resistance to *S*. Typhimurium in lethality and bacterial spread in this model (67, 68).

The natural microbiota of the mouse gut is able to outcompete *Salmonella* to occupy this ecological niche. Thus, a new model of typhlocolitis was developed employing streptomycin treatment to deplete commensal bacteria and to overcome the colonization resistance of mice. This infection model leads to a rapid induction of inflammation in cecum and colon (87, 88).

An influence of type I IFN signaling on the immune response to *S*. Typhimurium infection has been suggested during the analysis of *Usp18*-deficient mice (69, 89). USP18 is a deubiquinating protease with de-ISGylation activity specific for ISG15. It also limits JAK–STAT activation and is thus involved in the negative regulation of type I IFN signaling. During systemic *Salmonella* infection, increased STAT1 activation correlated with impaired STAT4 activation and reduced IFN-γ production, and *Usp18* mutant mice are more susceptible to systemic (i.e., typhoid) *S*. Typhimurium infection (89). By contrast, in the streptomycin-induced model of typhlocolitis, mutant *Usp18* mice display lower pathology scores, low IFN-γ production but upregulated type I IFN signaling compared to control mice, resulting in earlier systemic dissemination of the bacteria and decreased survival (69).

Interestingly, influenza-induced type I IFN suppress host intestinal immunity leading to increased susceptibility to secondary *Salmonella*-induced colitis (68). Influenza-induced type I IFN strongly inhibited the induction of antimicrobial and inflammatory genes such as IFN-γ, S100A9, and lipocalin-2 in response to secondary *S*. Typhimurium infection causing increased intestinal colonization and increased bacterial dissemination but reduced immunopathology (68).

In summary, these studies suggest a detrimental effect of type I IFN on the growth and dissemination of bacteria in *Salmonella*-induced typhlocolitis whereas it limits intestinal inflammation. These effects are more obivous when type I IFN production is boosted by influenza infection or polyI:C treatment (68). Additional studies are required to determine the direct and indirect effects of type I IFN on *S*. Typhimurium-induced typhlocolitis. Moreover, the role of type III IFN in this model has yet to be addressed.

### Other Enteric Bacterial Infections

The role of type I or III IFN in other enteric bacterial infections [e.g., enteropathogenic (EPEC) and enterohemorrhagic (EHEC) *Escherichia coli, Citrobacter rodentium, Campylobacter jejuni*, or *Yersinia enterocolitica*] has not been extensively addressed in *in vivo* infection models.

*Citrobacter rodentium* and EPEC developed immune evasion strategies targeting type I IFN signaling, suggesting a protective effect of type I IFN against those pathogens. *C. rodentium* has been reported to actively inhibit epithelial type I IFN production employing a mechanism depending on the type III secretion system (T3SS) (90). Furthermore, it also reduces type I IFN signaling by decreasing nuclear translocation of phosphorylated STAT1 even after co-stimulation with IFN-β (90).

Infection of a colon cancer cell line with EPEC results in a modest IFN-β production, which regulates tight junction proteins such as claudin 1 and occludin to maintain barrier function (91). Using a distinct mechanism to *C. rodentium*, an EPEC T3SS effector, NleD, reduces IFN-β induction by inhibition of RNase L, ultimately resulting in evasion of antibacterial activities and disruption of barrier function (91).

Type I IFN also play a protective role during oral *Y. enterocolitica* infection. TRIF-deficient mice are highly susceptible to *Y. enterocolitica* infection with increased bacterial spread and lethality. This could be prevented by IFN-β treatment of TRIF-deficient mice and IFNAR blocking of wild-type mice recapitulates increased bacterial burden (70).

*Campylobacter jejuni* infection of murine DCs leads to TLR4/TRIF-dependent activation of IRF-3 and secretion of type I IFN and cooperative signaling through both TLR4/MyD88 and TLR4/TRIF pathways is required for full Th1 priming ability (92). Interestingly, production of type I IFN and other cytokines in splenic tissue is significantly increased by lipo-oligosaccharide (LOS) sialylation (93).

Whether type I IFN and/or type III IFN responses to sialylated *C. jejuni* have a role in oral infection models of mice awaits further investigation. Recently, novel mouse models have been developed relying on the eradication or modification of the murine gut microbiota using broad-spectrum antibiotic treatment and subsequent re-association with a complex human microbiota. This approach leads to stable colonization with *C. jejuni* and a proinflammatory response in the colons of infected mice (94). Hopefully, these models will provide the necessary tools to answer the questions of an *in vivo* importance of type I IFN and/or type III IFN in the mucosal immune response to *C. jejuni*. This will be of particular importance since type I IFN responses have also been observed in former *C. jejuni*-infected patients suffering from Guillain–Barré syndrome (GBS), an autoimmune neuropathy where infection with *C. jejuni* is the most common predisposing factor (95). *C. jejuni* LOS activates TLR4 and high responsiveness of DCs isolated from former GBS patients was hypothesized to influence the development of GBS. Indeed, although a strong variability in DC responses to LOS was observed, the frequency of CD38, CD40, and type I IFN high responders was significantly increased in the *C. jejuni*-related former GBS patients compared to controls (95).

## Importance of Type I and III IFN Signaling for Intestinal Homeostasis

### A Role for Type I and III IFN in Shaping the Microbiota

Intestinal homeostasis is dependent on the tight interplay between the host and commensal flora. The flora directly protects the host from intestinal pathogens by competition for nutrients and space. Furthermore, commensal bacteria are important for the development of the immune system and maintenance of the barrier. The host immune system has also a great influence on the composition of the microbiota (96). Several studies have suggested that IFN signaling influences cross talk between the host and the flora.

It has long been recognized that the microbiota of different inbred mouse strains changes over time despite shared origin, which has been attributed to host genetic factors (97). Gene-expression analysis of the colonic mucosa revealed that IFN-responsive genes are differentially regulated between the different mouse strains and might thus contribute to microbiota regulation (97). Indeed, microbiota analysis of mice with selective ablation of type I IFN signaling in the intestinal epithelium revealed changes of the microbiota composition if littermates were housed separately for 8–12 weeks (76). However, it is not known whether this is a direct effect of type I IFN signaling by the epithelium and what the mechanistic relationship is.

The community stability of the gut microbiota might also depend on IFN signaling (98). IRF-9-deficient mice unable to respond to either type I IFN or type III IFN displayed a significantly higher temporal variation than wild-type mice, which was accompanied by an increased presence of T cells and neutrophils. However, STAT1-deficient mice, which classically are unable to respond to type I IFN, type III IFN, and IFN-γ, were not significantly different from wild-type mice implicating that a previously unrecognized pathway might be involved (98). Indeed, a role for IRF-9 in non-canonical IFN signaling and beyond the IFN response has been suggested (99).

Recently, Deriu et al. reported that systemic influenza-induced type I IFN production significantly alters the intestinal microbiota profile (68). While under their experimental conditions uninfected wild-type and IFNAR1-deficient mice displayed similar fecal microbial communities, influenza infection-induced type I IFN signaling resulted in a depletion of indigenous *segmented filamentous bacteria* and enhanced colonization with *Enterobacteriaceae* (68).

Type III IFN signaling on its own does not have a strong effect on the flora composition, as 16S rDNA sequencing of the V4 region of fecal pellets from wild-type or IFN-λR1^−/−^ mice revealed similar bacterial class composition (13). It is important to note that microbiota studies are difficult to control and generalizations from one specific study should be drawn carefully as major differences have been found between mouse facilities.

### A Role for Type I and III IFN in Shaping Intestinal Homeostasis

Upon colonization of germ-free mice with a two-component bacterial community, IFN-responsive genes are strongly upregulated in cecal epithelia (100).

Several studies have also demonstrated a role for commensal-induced tonic type I IFN signaling in the steady state to keep the host in a state of alertness against a systemic viral infection (101–103). Mononuclear phagocytes isolated from non-mucosal lymph nodes of germ-free mice are unable to upregulate type I IFN genes after stimulation with microbial ligands and thus fail to prime NK cells and antiviral immunity (102). A second study came to similar conclusions demonstrating decreased antiviral gene expression and defective ISG expression of macrophages from antibiotic-treated mice (101). The nature of the commensal bacterial species or microbial product responsible for the tonic type I IFN production is unknown and its identification remains a future challenge.

Recently, a connection between the increased susceptibility of liver cirrhosis patients to bacterial infections and tonic type I IFN signaling was made (81). In a murine model of liver fibrosis, translocated gut bacteria induced the expression of a tonic type I IFN signature in the liver, which in turn conditioned myeloid cells to produce vast amounts of type I IFN upon subsequent systemic infection with *L. monocytogenes*. Type I IFN signaling resulted in the production of IL-10 by myeloid cells, which hampered antibacterial immunity. Key findings of the murine model such as a type I IFN signature in cirrhotic livers and myeloid IL-10 production could also be recapitulated in human patient material. Thus, tonic type I IFN signaling induced by the translocated gut microbiota can also have detrimental effects (81).

Plasmacytoid dendritic cells are known to be important producers of type I IFN after viral infections and treatment with bacterial nucleic acids. Interestingly, pDCs derived from Peyers’s patches are incapable of producing significant amounts of type I IFN after stimulation with CpG-enriched oligodeoxynucleotides while producing IL-12 (104). Thus, although tonic type I IFN production by myeloid cells fulfils important functions to maintain basal levels of ISGs (81, 101, 102), the mucosal microenvironment prevents production of vast amounts of type I IFN by pDCs—presumably to prevent harmful immune responses to commensal microorganisms (104). Other sources of type I IFN in the GALT are stromal cells. Indeed stroma-derived type I IFN has been shown to induce APRIL and BAFF expression by pDCs, which facilitates T cell-independent IgA production by mucosal B cells (105).

Of note, the expression of ISGs at steady state seems to be driven by type I IFN and type III IFN in a cell type-specific manner. The cells of the lamina propria are mainly activated by type I IFN whereas IECs respond mainly to baseline levels of type III IFN due to low IFNAR expression on IECs, which is in accordance with the response pattern observed during viral infection (Figure 1) (14). Furthermore, the role of altered type I IFN signaling on IECs was addressed by several groups (50, 76, 106).

Tschurtschenthaler and colleagues reported that the lack of type I IFN signaling in IECs leads to a hyperproliferative phenotype (76). Particularly the secretory cell types Paneth and goblet cells are expanded in a setting where IECs are the only cells impaired in type I IFN signaling (Villin-Cre Ifnar^fl/fl^). Although this study suggests that type I IFN signaling occurs in IECs, the proliferative phenotype is secondary due to alterations of the microflora, as cohousing of the mice resolved the differences (76).

Katlinskaya et al. used a model of decreased IFNAR degradation in IECs to study type I IFN signaling outcome in IECs (107). CK1α can phosphorylate IFNAR1, which subsequently leads to its degradation (108). Genetic ablation of CK1α in IECs leads to increased IFNAR levels and elevated type I IFN signaling in the epithelium. CK1α deficiency also results in β-catenin activation, which leads to hyperproliferation of the epithelium and loss of barrier function where type I IFN signaling is additionally blocked. Elevated type I IFN signaling, however, inhibits β-catenin-driven proliferation and induces apoptosis maintaining barrier integrity (107).

In another model of chronically elevated levels of systemic type I IFN, epithelial cell turnover was increased in various tissues including the intestine (106). This effect was not due to direct signaling of type I IFN in epithelial cells but by induction of Apol9a/b in macrophages or stromal cells that subsequently promoted the turnover of epithelial cells (106).

Taken together, epithelial type I IFN signaling seems to have a pleiotropic effect depending on levels of receptor expression, ligand abundance, microflora, and tissue context.

## Importance of Type I/III IFN Signaling Under Inflammatory Conditions

Experiments employing different murine models of colitis such as dextran sodium sulfate (DSS) colitis or the T cell transfer model have provided a complex picture of the role of type I/III IFN in intestinal inflammation. Administration of DSS in the drinking water leads to disruption of the epithelial barrier and an inflammatory reaction to microbial patterns and food antigens. The T cell transfer model on the other hand relies on the transfer of naive CD4^+^ T cells into immune-deficient mice (e.g., RAG^−/−^ mice), which undergo activation and proliferation in response to microbial products to provoke inflammatory colitis when a suppressive T cell population (Foxp3^+^ Tregs) is absent (109, 110).

Katakura et al. investigated the role of type I IFN induction after administration of CpG ODN in experimental colitis (73). Mice deficient in type I IFN signaling are resistant to the CpG ODN-mediated effect and are more susceptible to DSS treatment than wild-type mice, suggesting a protective effect of type I IFN signaling.

By contrast, a therapeutic approach employing a transgenic *Lactobacillus acidophilus* strain constitutively expressing IFN-β (La-IFN-β) failed to protect against DSS-induced colitis but exacerbated the disease (74). Colitic mice pretreated with La-IFN-β displayed increased production of proinflammatory cytokines and decreased numbers of Tregs in their small intestine. *In vitro*, maturation of bone-marrow-derived DCs with La-IFN-β resulted in a threefold reduction of IFNAR1 and an impaired ability to induce Tregs (74). Thus, although a correlation between downregulation of IFNAR1 on DCs and exacerbation of colitis was observed, pretreatment of colitic mice with La-IFN-β surprisingly also resulted in increased intestinal damage (74). These results suggest that a tight regulation of type I IFN signaling is important for the balance of intestinal homeostasis.

The effect of type I IFN in experimental colitis might depend on the severity of inflammation and opposing roles in specific phases of intestinal damage and inflammation have been proposed (75, 111). At high DSS concentrations, type I IFN signaling protected against acute intestinal damage presumably by suppressing the release of IL-1β from colonic MHC class II^+^ cells (75). In the recovery phase after DSS treatment, type I IFN signaling resulted in delayed recovery from intestinal inflammation accompanied by increased cell apoptosis as well as an increase in chemokine production and subsequent infiltration of neutrophils and inflammatory monocytes (75). The potential of type I IFN signaling to either suppress acute colitis or delay the recovery might provide an explanation for the varying effects of type I IFN treatment on inflammatory bowel diseases (IBD) patients.

Several groups have analyzed the effect of type I IFN signaling on T cells (23, 71, 72, 112).

CD69 is suggested to be a regulator of intestinal homeostasis and is highly expressed on intestinal CD4^+^ T cells, which at steady state is driven by the microflora but can be further induced by type I IFN signaling (72, 113). Poly(I:C) treatment during T cell transfer colitis attenuates colitis by IFNAR-dependent CD69 induction on T cells, which leads to downregulation of proinflammatory cytokine levels (72).

Furthermore, Lee et al. identified a protective role of T cell type I IFN signaling by regulating the expression of Foxp3 and the suppressive effect of Tregs (71). Whereas Tregs isolated from wild-type mice suppressed colitis when cotransferred with naïve CD4^+^ T cells, the same cell population derived from IFNAR1^−/−^ mice failed to do so. Although Tregs undergo normal development in IFNAR1^−/−^ mice, the cells acquire a dysfunctional phenotype accompanied with reduced Foxp3 expression when cotransferred with naive CD4^+^ T cells into RAG1^−/−^ recipients. Administration of recombinant IFN-α ameliorated T-cell-dependent colitis by augmenting the number of Foxp3^+^ Tregs suggesting a potential therapeutic application of type I IFN in intestinal inflammation (71, 72, 113).

By contrast, several studies suggested an indirect effect of TLR9 ligands and type I IFN signaling for protection in T cell-mediated experimental colitis (23, 99, 112). While a role for B cells was excluded, colitis-reducing effects of CpG ODN were mediated by CD11c^+^ cells and required functional type I IFN signaling in a model of T cell transfer colitis (112).

Kole et al. showed that colon mononuclear phagocytes deficient in type I IFN signaling failed to produce regulatory cytokines such as IL-10, IL-27, and IL-1RA in response to TLR activation. Furthermore in the T cell transfer colitis model, IFNAR signaling of host hematopoietic cells was important to limit effector cell expansion and to promote the stabilization of Foxp3^+^ Tregs (23).

Intestinal epithelial cells and in particular Paneth cells have also been proposed to be a target of type I IFN signaling in the intestine (76, 114). Paneth cells are epithelial cells located at the bases of small-intestinal crypts specialized in secretion of antimicrobial peptides and factors to sustain epithelial stem cells and progenitor cells (115). Mice with a specific deletion of IFNAR1 in IECs display expansion of Paneth cell numbers and epithelial hyperproliferation when compared with wild-type littermates. Although epithelial-specific deletion of IFNAR1 did not impact on the severity of spontaneous or DSS-induced intestinal inflammation, they exhibited increased tumor burden in the azoxymethane/DSS model of colitis-associated colon cancer (76), Both spontaneous epithelial hyperproliferation and tumor promotion are dependent on the microbial flora, since differences between wild-type and IEC-specific IFNAR1-deficient mice were only apparent if the mice were housed separately (76).

Human and murine IECs display high responsiveness to type III IFN treatment (12, 116). A recent study has demonstrated a protective role of type III IFN signaling in DSS-induced colitis (77). Mice deficient for the type III IFN receptor lost significantly more weight and suffered from significantly increased intestinal damage after DSS treatment when compared to WT controls. Additional loss of the type I IFN receptor did not change the pathology scores compared to the single loss of type III IFN receptor, emphasizing a prominent role of type III IFN signaling in this model. The protective effect of type III IFN signaling is independent of potential changes in the microbiota since the same results were obtained when wild-type and type III IFN signaling-deficient mice were cohoused for 3 weeks (77).

## Type I IFN and IBD

The IBD, comprising Crohn’s disease (CD), and ulcerative colitis (UC) are chronic debilitating inflammatory disorders of the gastrointestinal tract. IBD affects about 0.2% of Western populations and there is no current cure, typically requiring long-term treatment with immune suppressive agents and, in many cases, surgical intervention. Although the etiology remains unclear, IBD is thought to arise due to aberrant immune responses to components of the commensal bacterial microbiota (117). Recent genome-wide association studies have identified more than 160 genetic susceptibility loci for IBD, with affected genes involved in immunity and in barrier function (118). Many of those single-nucleotide polymorphisms (SNPs) are found in genes associated with pathogenic cytokine circuits, such as the Th17/IL23 circuit, IL-10, and type I IFN-I signaling (119). The majority of signaling mediators are shared between different cytokine signaling cascades and therefore exact determination of the relevant pathways is impossible from the genetic data only. Interestingly, several of the IBD-associated genes are also involved in the type I IFN signaling pathway. The rs2284553 SNP is commonly associated with the *IFNGR2* gene but could also affect the *IFNAR1* gene (118). *JAK2, TYK2, STAT1*, and *STAT3* genes harbor identified SNPs and are signaling mediators in many cytokine pathways such as IL-22, IL-10, and also type I/III IFN. Furthermore, polymorphisms in the *MDA5* or *IRF5* gene might alter the production of type I/III IFN (118). Although the type I IFN signaling network is not one of the major players in IBD pathology, slight alterations may contribute to the imbalanced immune response at the lamina propria, as suggested by mouse studies.

Indeed, Giles and colleagues analyzed the responsiveness of T cells from healthy controls and IBD patients to IFN-β and found that IFN-β signaling modulates colonic T cell responses in a context-dependent manner. Human colonic T cells were responsive to exogenous IFN-β and endogenous IFN-β influenced the cytokine profile of *ex vivo* cultured T cells. T cells from healthy controls produced decreased levels of IL-10 in the absence of IFN-β signaling whereas T cells from IBD patients produced elevated levels of proinflammatory cytokines (120).

Interferons-β has been approved for the treatment of multiple sclerosis (MS); however, a subset of patients does not respond to the treatment. Axtell and colleagues analyzed the effect of IFN-β on different Th subsets and found that IFN-β treatment in a mouse model of EAE attenuates disease development in a Th1-driven pathology, but had no effect or even exacerbates pathology in Th17-driven disease. Furthermore, they could correlate high IL-17-F serum levels in MS patients to non-responsiveness toward IFN-β treatment. These findings confirm the immunomodulatory role of IFN-β but also demonstrate the diverse consequences it has in different context with opposing effects within a Th1 and Th17 setting (121).

Despite the varying results from mouse studies on the role of type I IFN in colitis and the discrepancy between type I IFN effects on suppressing acute colitis and delaying recovery (74, 75), type I IFN have been suggested for the treatment of IBD. Several small studies have evaluated the consequences of IFN-β1a in IBD patients with varying results (122–128). Although small pilot studies suggested a beneficial outcome of type I IFN treatment of IBD patients (123, 124), a placebo-controlled, double-blind study on Crohn’s patients in remission did not find any improvement by IFN-β1a treatment on the maintenance of remission (127). Also two randomized placebo-controlled studies in UC patients with active disease could not show a beneficial effect of IFN-α or IFN-β1 treatment on disease remission (125, 126). A small study analyzing cytokine levels before and after treatment with IFN-β1a found a correlation between responsiveness and reduction of IL-13 levels in UC patients. The unresponsiveness to IFN-β1 treatment correlated with elevated levels of IL-17 in accordance with the findings in MS patients (121, 128).

Taken together, these studies do not support a beneficial outcome of type I IFN treatment during IBD. This conclusion was also drawn in a recent intervention review analyzing all trial data published on the effectiveness of type I IFN treatment on remission in UC patients (129). However, considering the analysis of IFN-β non-responsiveness of patients with MS (121), context-specific responsiveness of T cells toward type I IFN (120), and controversial findings in mouse studies (23, 74, 75), the effect of the treatment might vary between Th profiles of patients and a careful pre-selection of patients would be required. Further studies with sufficient patient numbers and thorough analysis of immunological and disease parameters are required.

## Type I IFN and Celiac Disease

Celiac disease is a small-intestinal enteropathy characterized by an aberrant T cell-mediated immune response of susceptible individuals to dietary gluten. The pathogenic adaptive immune response is initiated by the interplay between gluten and the MHC class II molecules HLA-DQ2 and DQ8 and is characterized by a potent Th1 response. The excessive tissue destruction is further driven by a severe IEL hyperplasia targeting IECs. Histologically, celiac disease is characterized by villous flattening, crypt hyperplasia, and IEL infiltration. Affected individuals can present with very variable symptoms ranging from asymptomatic to severe symptoms ascribed to impaired absorption of nutrients (130).

The strongest genetic factor for the disease is HLA-DQ2 and DQ8; however, it is now recognized that further immune-regulatory or activating factors are required for disease establishment. Several celiac disease susceptibility loci in genes associated with innate immune responses have been identified, suggesting a role for innate immunity in the development of the disease (131).

An influence of type I IFN on the development of celiac disease has been widely discussed. Indeed, a number of case studies reported on the development of diarrhea and the onset of celiac disease during treatment with IFN-α for chronic hepatitis C patients (132–137). A retrospective study of 534 hepatitis C patients with or without symptoms of celiac disease showed an activation of silent celiac disease in the majority of patients positive for transglutaminase antibodies while on IFN therapy (138). The immunomodulatory properties of type I IFN might worsen underlying autoimmune disorders and monitoring of hepatitis C patients for celiac disease before starting an IFN therapy has been suggested. A potential role of cotreatment with ribavirin, which promotes a Th1-mediated immune response while suppressing Th2 responses, has also been discussed (133).

The high prevalence of celiac disease in HCV patients treated with IFN-α was investigated in a study including 210 chronic hepatitis C patients. This study failed to detect a significant association of celiac disease and HCV infection and in addition came to the conclusion that IFN-α therapy *per se* does not trigger celiac disease in patients negative for endomysium (EMA) and tissue transglutaminase (139). It does not however rule out that IFN-α treatment might trigger the development of celiac disease in susceptible individuals.

To investigate the underlying mechanisms, explant cultures of human fetal gut were analyzed after activation of T cells with anti-CD3 and IFN-α. While single treatment with either anti-CD3 or IFN-α alone did not trigger any profound changes, the combination of both resulted in enhanced Th1 responses and crypt cell hyperplasia associated with enhanced STAT1, STAT3, and FYN phosphorylation. IFN-α treatment might thus facilitate activation of Th1-reactive cells and trigger immunopathology (135, 140).

Onset of celiac disease-like symptoms have also been observed in a case of chronic myeloid leukemia treated with IFN-α again suggesting a role of type I IFN in promoting Th1 responses to gluten (135). Also, IFN-α protein was detected in duodenal tissue of celiac disease patients but not in control samples (135).

Further studies are required to determine whether a direct link exists between type I IFN signaling and celiac disease.

## Concluding Remarks

Although important progress has been made in recent years, additional studies are required to deepen our understanding of the role of type I IFN and type III IFN in the gut. Type I IFN signaling in enteric viral infections is mostly protective, whereas it can be detrimental in certain enteric bacterial infections. The clinical data using type I IFN for colitis treatment are partly contradictory and a larger number of patients are required to obtain conclusive results.

With the exception of some viral infection and colitis models, where type III IFN signaling is mostly protective, the data on illuminating the role of type III IFN signaling in the gut are generally scarce. It is now well established that epithelial cells are especially responsive to type III IFN, which enforces the mucosal barrier and prevents viral entry and infection. Less clear is the non-responsiveness of the epithelium to type I IFN observed under several conditions (Figure 1). What benefit do epithelial cells gain from the decreased responsiveness? Can receptor expression levels and localization fully explain reduced type I IFN responsiveness in the epithelium?

The narrow range of cells responding to type III IFN makes it an attractive target for future clinical studies with increased specificity and fewer side effects than type I IFN.

## Author Contributions

JP and SST wrote the manuscript.

## Conflict of Interest Statement

The authors declare that the research was conducted in the absence of any commercial or financial relationships that could be construed as a potential conflict of interest.

## References

[1] Pott J Hornef M. Innate immune signalling at the intestinal epithelium in homeostasis and disease. EMBO Rep (2012) 13(8):684–98. 10.1038/embor.2012.96 22801555 PMC3410395

[2] Hooper LV. Epithelial cell contributions to intestinal immunity. Adv Immunol (2015) 126:129–72. 10.1016/bs.ai.2014.11.003 25727289

[3] Decker T Muller M Stockinger S. The yin and yang of type I interferon activity in bacterial infection. Nat Rev Immunol (2005) 5(9):675–87. 10.1038/nri1684 16110316

[4] Stockinger S Decker T. Novel functions of type I interferons revealed by infection studies with *Listeria monocytogenes*. Immunobiology (2008) 213(9–10):889–97. 10.1016/j.imbio.2008.07.020 18926303

[5] McNab F Mayer-Barber K Sher A Wack A O’Garra A. Type I interferons in infectious disease. Nat Rev Immunol (2015) 15(2):87–103. 10.1038/nri3787 25614319 PMC7162685

[6] Stifter SA Feng CG. Interfering with immunity: detrimental role of type I IFNs during infection. J Immunol (2015) 194(6):2455–65. 10.4049/jimmunol.1402794 25747907

[7] Kotenko SV Gallagher G Baurin VV Lewis-Antes A Shen M Shah NK IFN-lambdas mediate antiviral protection through a distinct class II cytokine receptor complex. Nat Immunol (2003) 4(1):69–77. 10.1038/ni875 12483210

[8] Sheppard P Kindsvogel W Xu W Henderson K Schlutsmeyer S Whitmore TE IL-28, IL-29 and their class II cytokine receptor IL-28R. Nat Immunol (2003) 4(1):63–8. 10.1038/ni873 12469119

[9] Wack A Terczynska-Dyla E Hartmann R. Guarding the frontiers: the biology of type III interferons. Nat Immunol (2015) 16(8):802–9. 10.1038/ni.3212 26194286 PMC7096991

[10] Mordstein M Kochs G Dumoutier L Renauld JC Paludan SR Klucher K Interferon-lambda contributes to innate immunity of mice against influenza A virus but not against hepatotropic viruses. PLoS Pathog (2008) 4(9):e1000151. 10.1371/journal.ppat.1000151 18787692 PMC2522277

[11] Mordstein M Neugebauer E Ditt V Jessen B Rieger T Falcone V Lambda interferon renders epithelial cells of the respiratory and gastrointestinal tracts resistant to viral infections. J Virol (2010) 84(11):5670–7. 10.1128/JVI.00272-10 20335250 PMC2876583

[12] Pott J Mahlakoiv T Mordstein M Duerr CU Michiels T Stockinger S IFN-lambda determines the intestinal epithelial antiviral host defense. Proc Natl Acad Sci U S A (2011) 108(19):7944–9. 10.1073/pnas.1100552108 21518880 PMC3093475

[13] Baldridge MT Nice TJ McCune BT Yokoyama CC Kambal A Wheadon M Commensal microbes and interferon-lambda determine persistence of enteric murine norovirus infection. Science (2015) 347(6219):266–9. 10.1126/science.1258025 25431490 PMC4409937

[14] Mahlakoiv T Hernandez P Gronke K Diefenbach A Staeheli P Leukocyte-derived IFN-alpha/beta and epithelial IFN-lambda constitute a compartmentalized mucosal defense system that restricts enteric virus infections. PLoS Pathog (2015) 11(4):e1004782 10.1371/journal.ppat.1004782 25849543 PMC4388470

[15] Nice TJ Baldridge MT McCune BT Norman JM Lazear HM Artyomov M Interferon-lambda cures persistent murine norovirus infection in the absence of adaptive immunity. Science (2015) 347(6219):269–73. 10.1126/science.1258100 25431489 PMC4398891

[16] Lazear HM Nice TJ Diamond MS Interferon-lambda: immune functions at barrier surfaces and beyond. Immunity (2015) 43(1):15–28. 10.1016/j.immuni.2015.07.001 26200010 PMC4527169

[17] Odendall C Dixit E Stavru F Bierne H Franz KM Durbin AF Diverse intracellular pathogens activate type III interferon expression from peroxisomes. Nat Immunol (2014) 15(8):717–26. 10.1038/ni.2915 24952503 PMC4106986

[18] Iversen MB Paludan SR. Mechanisms of type III interferon expression. J Interferon Cytokine Res (2010) 30(8):573–8. 10.1089/jir.2010.0063 20645874

[19] Onoguchi K Yoneyama M Takemura A Akira S Taniguchi T Namiki H Viral infections activate types I and III interferon genes through a common mechanism. J Biol Chem (2007) 282(10):7576–81. 10.1074/jbc.M608618200 17204473

[20] Osterlund PI Pietila TE Veckman V Kotenko SV Julkunen I. IFN regulatory factor family members differentially regulate the expression of type III IFN (IFN-lambda) genes. J Immunol (2007) 179(6):3434–42. 10.4049/jimmunol.179.6.3434 17785777

[21] Thomson SJ Goh FG Banks H Krausgruber T Kotenko SV Foxwell BM The role of transposable elements in the regulation of IFN-lambda1 gene expression. Proc Natl Acad Sci U S A (2009) 106(28):11564–9. 10.1073/pnas.0904477106 19570999 PMC2710658

[22] McKenna K Beignon AS Bhardwaj N Plasmacytoid dendritic cells: linking innate and adaptive immunity. J Virol (2005) 79(1):17–27. 10.1128/JVI.79.1.17-27.2005 15596797 PMC538703

[23] Kole A He J Rivollier A Silveira DD Kitamura K Maloy KJ Type I IFNs regulate effector and regulatory T cell accumulation and anti-inflammatory cytokine production during T cell-mediated colitis. J Immunol (2013) 191(5):2771–9. 10.4049/jimmunol.1301093 23913971 PMC3896249

[24] Swiecki M Colonna M. The multifaceted biology of plasmacytoid dendritic cells. Nat Rev Immunol (2015) 15(8):471–85. 10.1038/nri3865 26160613 PMC4808588

[25] Stockinger S Kastner R Kernbauer E Pilz A Westermayer S Reutterer B Characterization of the interferon-producing cell in mice infected with *Listeria monocytogenes*. PLoS Pathog (2009) 5(3):e1000355. 10.1371/journal.ppat.1000355 19325882 PMC2654726

[26] Dresing P Borkens S Kocur M Kropp S Scheu S A fluorescence reporter model defines “Tip-DCs” as the cellular source of interferon beta in murine listeriosis. PLoS One (2010) 5(12):e15567 10.1371/journal.pone.0015567 21179567 PMC3002951

[27] Solodova E Jablonska J Weiss S Lienenklaus S Production of IFN-beta during *Listeria monocytogenes* infection is restricted to monocyte/macrophage lineage. PLoS One (2011) 6(4):e18543 10.1371/journal.pone.0018543 21494554 PMC3073975

[28] Chirdo FG Millington OR Beacock-Sharp H Mowat AM. Immunomodulatory dendritic cells in intestinal lamina propria. Eur J Immunol (2005) 35(6):1831–40. 10.1002/eji.200425882 16010704

[29] Hernandez PP Mahlakoiv T Yang I Schwierzeck V Nguyen N Guendel F Interferon-lambda and interleukin 22 act synergistically for the induction of interferon-stimulated genes and control of rotavirus infection. Nat Immunol (2015) 16(7):698–707. 10.1038/ni.3180 26006013 PMC4589158

[30] Swamy M Abeler-Dorner L Chettle J Mahlakoiv T Goubau D Chakravarty P Intestinal intraepithelial lymphocyte activation promotes innate antiviral resistance. Nat Commun (2015) 6:7090. 10.1038/ncomms8090 25987506 PMC4479038

[31] Wolk K Witte K Witte E Raftery M Kokolakis G Philipp S IL-29 is produced by T(H)17 cells and mediates the cutaneous antiviral competence in psoriasis. Sci Transl Med (2013) 5(204):204ra129. 10.1126/scitranslmed.3006245 24068736

[32] Monroe KM McWhirter SM Vance RE. Induction of type I interferons by bacteria. Cell Microbiol (2010) 12(7):881–90. 10.1111/j.1462-5822.2010.01478.x 20482555 PMC2897911

[33] Boxx GM Cheng G. The roles of type I interferon in bacterial infection. Cell Host Microbe (2016) 19(6):760–9. 10.1016/j.chom.2016.05.016 27281568 PMC5847370

[34] Lebreton A Lakisic G Job V Fritsch L Tham TN Camejo A A bacterial protein targets the BAHD1 chromatin complex to stimulate type III interferon response. Science (2011) 331(6022):1319–21. 10.1126/science.1200120 21252314

[35] Bierne H Travier L Mahlakoiv T Tailleux L Subtil A Lebreton A Activation of type III interferon genes by pathogenic bacteria in infected epithelial cells and mouse placenta. PLoS One (2012) 7(6):e39080. 10.1371/journal.pone.0039080 22720036 PMC3375250

[36] Pietila TE Latvala S Osterlund P Julkunen I. Inhibition of dynamin-dependent endocytosis interferes with type III IFN expression in bacteria-infected human monocyte-derived DCs. J Leukoc Biol (2010) 88(4):665–74. 10.1189/jlb.1009651 20610797

[37] Makela SM Osterlund P Julkunen I TLR ligands induce synergistic interferon-beta and interferon-lambda1 gene expression in human monocyte-derived dendritic cells. Mol Immunol (2011) 48(4):505–15. 10.1016/j.molimm.2010.10.005 21040977

[38] Schneider WM Chevillotte MD Rice CM. Interferon-stimulated genes: a complex web of host defenses. Annu Rev Immunol (2014) 32:513–45. 10.1146/annurev-immunol-032713-120231 24555472 PMC4313732

[39] Sommereyns C Paul S Staeheli P Michiels T. IFN-lambda (IFN-lambda) is expressed in a tissue-dependent fashion and primarily acts on epithelial cells in vivo. PLoS Pathog (2008) 4(3):e1000017. 10.1371/journal.ppat.1000017 18369468 PMC2265414

[40] Pulverer JE Rand U Lienenklaus S Kugel D Zietara N Kochs G Temporal and spatial resolution of type I and III interferon responses in vivo. J Virol (2010) 84(17):8626–38. 10.1128/JVI.00303-10 20573823 PMC2919002

[41] Souza-Fonseca-Guimaraes F Young A Mittal D Martinet L Bruedigam C Takeda K NK cells require IL-28R for optimal in vivo activity. Proc Natl Acad Sci U S A (2015) 112(18):E2376–84. 10.1073/pnas.1424241112 25901316 PMC4426428

[42] Blazek K Eames HL Weiss M Byrne AJ Perocheau D Pease JE IFN-lambda resolves inflammation via suppression of neutrophil infiltration and IL-1beta production. J Exp Med (2015) 212(6):845–53. 10.1084/jem.20140995 25941255 PMC4451128

[43] de Groen RA Groothuismink ZM Liu BS Boonstra A IFN-lambda is able to augment TLR-mediated activation and subsequent function of primary human B cells. J Leukoc Biol (2015) 98(4):623–30. 10.1189/jlb.3A0215-041RR 26130701

[44] Decker T Stockinger S Karaghiosoff M Muller M Kovarik P IFNs and STATs in innate immunity to microorganisms. J Clin Invest (2002) 109(10):1271–7. 10.1172/JCI15770 12021240 PMC150987

[45] Muller U Steinhoff U Reis LF Hemmi S Pavlovic J Zinkernagel RM Functional role of type I and type II interferons in antiviral defense. Science (1994) 264(5167):1918–21. 10.1126/science.8009221 8009221

[46] Bartlett NW Buttigieg K Kotenko SV Smith GL. Murine interferon lambdas (type III interferons) exhibit potent antiviral activity in vivo in a poxvirus infection model. J Gen Virol (2005) 86(Pt 6):1589–96. 10.1099/vir.0.80904-0 15914836

[47] Ank N West H Bartholdy C Eriksson K Thomsen AR Paludan SR. Lambda interferon (IFN-lambda), a type III IFN, is induced by viruses and IFNs and displays potent antiviral activity against select virus infections in vivo. J Virol (2006) 80(9):4501–9. 10.1128/JVI.80.9.4501-4509.2006 16611910 PMC1472004

[48] Nice TJ Osborne LC Tomov VT Artis D Wherry EJ Virgin HW Type I interferon receptor deficiency in dendritic cells facilitates systemic murine norovirus persistence despite enhanced adaptive immunity. PLoS Pathog (2016) 12(6):e1005684 10.1371/journal.ppat.1005684 27327515 PMC4915689

[49] Feng N Kim B Fenaux M Nguyen H Vo P Omary MB Role of interferon in homologous and heterologous rotavirus infection in the intestines and extraintestinal organs of suckling mice. J Virol (2008) 82(15):7578–90. 10.1128/JVI.00391-08 18495762 PMC2493311

[50] Lin JD Feng N Sen A Balan M Tseng HC McElrath C Distinct roles of type I and type III interferons in intestinal immunity to homologous and heterologous rotavirus infections. PLoS Pathog (2016) 12(4):e1005600. 10.1371/journal.ppat.1005600 27128797 PMC4851417

[51] Karst SM Wobus CE Lay M Davidson J Virgin HW. STAT1-dependent innate immunity to a Norwalk-like virus. Science (2003) 299(5612):1575–8. 10.1126/science.1077905 12624267

[52] Wang P Zhu S Yang L Cui S Pan W Jackson R Nlrp6 regulates intestinal antiviral innate immunity. Science (2015) 350(6262):826–30. 10.1126/science.aab3145 26494172 PMC4927078

[53] Angel J Franco MA Greenberg HB Bass D. Lack of a role for type I and type II interferons in the resolution of rotavirus-induced diarrhea and infection in mice. J Interferon Cytokine Res (1999) 19(6):655–9. 10.1089/107999099313802 10433367

[54] Karst SM Wobus CE Goodfellow IG Green KY Virgin HW. Advances in norovirus biology. Cell Host Microbe (2014) 15(6):668–80. 10.1016/j.chom.2014.05.015 24922570 PMC4113907

[55] Wobus CE Karst SM Thackray LB Chang KO Sosnovtsev SV Belliot G Replication of norovirus in cell culture reveals a tropism for dendritic cells and macrophages. PLoS Biol (2004) 2(12):e432. 10.1371/journal.pbio.0020432 15562321 PMC532393

[56] Gonzalez-Hernandez MB Liu T Payne HC Stencel-Baerenwald JE Ikizler M Yagita H Efficient norovirus and reovirus replication in the mouse intestine requires microfold (M) cells. J Virol (2014) 88(12):6934–43. 10.1128/JVI.00204-14 24696493 PMC4054386

[57] Changotra H Jia Y Moore TN Liu G Kahan SM Sosnovtsev SV Type I and type II interferons inhibit the translation of murine norovirus proteins. J Virol (2009) 83(11):5683–92. 10.1128/JVI.00231-09 19297466 PMC2681988

[58] Hwang S Maloney NS Bruinsma MW Goel G Duan E Zhang L Nondegradative role of Atg5-Atg12/Atg16L1 autophagy protein complex in antiviral activity of interferon gamma. Cell Host Microbe (2012) 11(4):397–409. 10.1016/j.chom.2012.03.002 22520467 PMC3348177

[59] Thackray LB Duan E Lazear HM Kambal A Schreiber RD Diamond MS Critical role for interferon regulatory factor 3 (IRF-3) and IRF-7 in type I interferon-mediated control of murine norovirus replication. J Virol (2012) 86(24):13515–23. 10.1128/JVI.01824-12 23035219 PMC3503103

[60] Johansson C Wetzel JD He J Mikacenic C Dermody TS Kelsall BL. Type I interferons produced by hematopoietic cells protect mice against lethal infection by mammalian reovirus. J Exp Med (2007) 204(6):1349–58. 10.1084/jem.20061587 17502662 PMC2118611

[61] Carrero JA. Confounding roles for type I interferons during bacterial and viral pathogenesis. Int Immunol (2013) 25(12):663–9. 10.1093/intimm/dxt050 24158954 PMC3839063

[62] Auerbuch V Brockstedt DG Meyer-Morse N O’Riordan M Portnoy DA. Mice lacking the type I interferon receptor are resistant to *Listeria monocytogenes*. J Exp Med (2004) 200(4):527–33. 10.1084/jem.20040976 15302899 PMC2211930

[63] Carrero JA Calderon B Unanue ER Listeriolysin O from *Listeria monocytogenes* is a lymphocyte apoptogenic molecule. J Immunol (2004) 172(8):4866–74. 10.4049/jimmunol.172.8.4866 15067065

[64] O’Connell RM Saha SK Vaidya SA Bruhn KW Miranda GA Zarnegar B Type I interferon production enhances susceptibility to *Listeria monocytogenes* infection. J Exp Med (2004) 200(4):437–45. 10.1084/jem.20040712 15302901 PMC2211937

[65] Kernbauer E Maier V Rauch I Muller M Decker T Route of infection determines the impact of type I interferons on innate immunity to *Listeria monocytogenes*. PLoS One (2013) 8(6):e65007 10.1371/journal.pone.0065007 23840314 PMC3686784

[66] Pitts MG Myers-Morales T D’Orazio SE Type I IFN does not promote susceptibility to foodborne *Listeria monocytogenes*. J Immunol (2016) 196(7):3109–16. 10.4049/jimmunol.1502192 26895837 PMC4799772

[67] Perkins DJ Rajaiah R Tennant SM Ramachandran G Higginson EE Dyson TN *Salmonella* Typhimurium co-opts the host type I IFN system to restrict macrophage innate immune transcriptional responses selectively. J Immunol (2015) 195(5):2461–71. 10.4049/jimmunol.1500105 26202980 PMC4546913

[68] Deriu E Boxx GM He X Pan C Benavidez SD Cen L Influenza virus affects intestinal microbiota and secondary *Salmonella* infection in the gut through type I interferons. PLoS Pathog (2016) 12(5):e1005572. 10.1371/journal.ppat.1005572 27149619 PMC4858270

[69] Richer E Yuki KE Dauphinee SM Lariviere L Paquet M Malo D. Impact of Usp18 and IFN signaling in *Salmonella*-induced typhlitis. Genes Immun (2011) 12(7):531–43. 10.1038/gene.2011.38 21614019

[70] Sotolongo J Espana C Echeverry A Siefker D Altman N Zaias J Host innate recognition of an intestinal bacterial pathogen induces TRIF-dependent protective immunity. J Exp Med (2011) 208(13):2705–16. 10.1084/jem.20110547 22124111 PMC3244044

[71] Lee SE Li X Kim JC Lee J Gonzalez-Navajas JM Hong SH Type I interferons maintain Foxp3 expression and T-regulatory cell functions under inflammatory conditions in mice. Gastroenterology (2012) 143(1):145–54. 10.1053/j.gastro.2012.03.042 22475534 PMC3729390

[72] Radulovic K Manta C Rossini V Holzmann K Kestler HA Wegenka UM CD69 regulates type I IFN-induced tolerogenic signals to mucosal CD4 T cells that attenuate their colitogenic potential. J Immunol (2012) 188(4):2001–13. 10.4049/jimmunol.1100765 22250092

[73] Katakura K Lee J Rachmilewitz D Li G Eckmann L Raz E. Toll-like receptor 9-induced type I IFN protects mice from experimental colitis. J Clin Invest (2005) 115(3):695–702. 10.1172/JCI22996 15765149 PMC1051992

[74] McFarland AP Savan R Wagage S Addison A Ramakrishnan K Karwan M Localized delivery of interferon-beta by *Lactobacillus* exacerbates experimental colitis. PLoS One (2011) 6(2):e16967 10.1371/journal.pone.0016967 21365015 PMC3041828

[75] Rauch I Hainzl E Rosebrock F Heider S Schwab C Berry D Type I interferons have opposing effects during the emergence and recovery phases of colitis. Eur J Immunol (2014) 44(9):2749–60. 10.1002/eji.201344401 24975266

[76] Tschurtschenthaler M Wang J Fricke C Fritz TM Niederreiter L Adolph TE Type I interferon signalling in the intestinal epithelium affects Paneth cells, microbial ecology and epithelial regeneration. Gut (2014) 63(12):1921–31. 10.1136/gutjnl-2013-305863 24555997

[77] Rauch I Rosebrock F Hainzl E Heider S Majoros A Wienerroither S Noncanonical effects of IRF9 in intestinal inflammation: more than type I and type III interferons. Mol Cell Biol (2015) 35(13):2332–43. 10.1128/MCB.01498-14 25918247 PMC4456449

[78] Carrero JA Calderon B Unanue ER Type I interferon sensitizes lymphocytes to apoptosis and reduces resistance to *Listeria* infection. J Exp Med (2004) 200(4):535–40. 10.1084/jem.20040769 15302900 PMC2211931

[79] Zwaferink H Stockinger S Reipert S Decker T. Stimulation of inducible nitric oxide synthase expression by beta interferon increases necrotic death of macrophages upon *Listeria monocytogenes* infection. Infect Immun (2008) 76(4):1649–56. 10.1128/IAI.01251-07 18268032 PMC2292882

[80] Carrero JA Calderon B Unanue ER. Lymphocytes are detrimental during the early innate immune response against *Listeria monocytogenes*. J Exp Med (2006) 203(4):933–40. 10.1084/jem.20060045 16549598 PMC2118284

[81] Hackstein CP Assmus LM Welz M Klein S Schwandt T Schultze J Gut microbial translocation corrupts myeloid cell function to control bacterial infection during liver cirrhosis. Gut (2016) 66:507–18. 10.1136/gutjnl-2015-311224 27432540

[82] Rayamajhi M Humann J Penheiter K Andreasen K Lenz LL. Induction of IFN-alphabeta enables *Listeria monocytogenes* to suppress macrophage activation by IFN-gamma. J Exp Med (2010) 207(2):327–37. 10.1084/jem.20091746 20123961 PMC2822610

[83] Cossart P. Illuminating the landscape of host-pathogen interactions with the bacterium *Listeria monocytogenes*. Proc Natl Acad Sci U S A (2011) 108(49):19484–91. 10.1073/pnas.1112371108 22114192 PMC3241796

[84] Disson O Grayo S Huillet E Nikitas G Langa-Vives F Dussurget O Conjugated action of two species-specific invasion proteins for fetoplacental listeriosis. Nature (2008) 455(7216):1114–8. 10.1038/nature07303 18806773

[85] Archambaud C Nahori MA Soubigou G Becavin C Laval L Lechat P Impact of lactobacilli on orally acquired listeriosis. Proc Natl Acad Sci U S A (2012) 109(41):16684–9. 10.1073/pnas.1212809109 23012479 PMC3478606

[86] Valdez Y Ferreira RB Finlay BB Molecular mechanisms of *Salmonella* virulence and host resistance. Curr Top Microbiol Immunol (2009) 337:93–127. 10.1007/978-3-642-01846-6_4 19812981

[87] Barthel M Hapfelmeier S Quintanilla-Martinez L Kremer M Rohde M Hogardt M Pretreatment of mice with streptomycin provides a *Salmonella enterica* serovar Typhimurium colitis model that allows analysis of both pathogen and host. Infect Immun (2003) 71(5):2839–58. 10.1128/IAI.71.5.2839-2858.2003 12704158 PMC153285

[88] Stecher B Paesold G Barthel M Kremer M Jantsch J Stallmach T Chronic *Salmonella enterica* serovar Typhimurium-induced colitis and cholangitis in streptomycin-pretreated Nramp1+/+ mice. Infect Immun (2006) 74(9):5047–57. 10.1128/IAI.00072-06 16926396 PMC1594839

[89] Richer E Prendergast C Zhang DE Qureshi ST Vidal SM Malo D N-ethyl-N-nitrosourea-induced mutation in ubiquitin-specific peptidase 18 causes hyperactivation of IFN-alphass signaling and suppresses STAT4-induced IFN-gamma production, resulting in increased susceptibility to *Salmonella typhimurium*. J Immunol (2010) 185(6):3593–601. 10.4049/jimmunol.1000890 20693420 PMC3987673

[90] Gao X Pham TH Feuerbacher LA Chen K Hays MP Singh G Citrobacter rodentium NleB inhibits tumor necrosis factor (TNF) receptor-associated factor 3 (TRAF3) ubiquitination to reduce host type I interferon production. J Biol Chem (2016) 291:18232–8. 10.1074/jbc.M116.738278 27387501 PMC5000071

[91] Long TM Nisa S Donnenberg MS Hassel BA. Enteropathogenic *Escherichia coli* inhibits type I interferon- and RNase L-mediated host defense to disrupt intestinal epithelial cell barrier function. Infect Immun (2014) 82(7):2802–14. 10.1128/IAI.00105-14 24733098 PMC4097611

[92] Rathinam VA Vanaja SK Waggoner L Sokolovska A Becker C Stuart LM TRIF licenses caspase-11-dependent NLRP3 inflammasome activation by gram-negative bacteria. Cell (2012) 150(3):606–19. 10.1016/j.cell.2012.07.007 22819539 PMC3660860

[93] Huizinga R Easton AS Donachie AM Guthrie J van Rijs W Heikema A Sialylation of *Campylobacter jejuni* lipo-oligosaccharides: impact on phagocytosis and cytokine production in mice. PLoS One (2012) 7(3):e34416. 10.1371/journal.pone.0034416 22470569 PMC3314637

[94] Bereswill S Fischer A Plickert R Haag LM Otto B Kuhl AA Novel murine infection models provide deep insights into the “menage a trois” of *Campylobacter jejuni*, microbiota and host innate immunity. PLoS One (2011) 6(6):e20953 10.1371/journal.pone.0020953 21698299 PMC3115961

[95] Huizinga R van den Berg B van Rijs W Tio-Gillen AP Fokkink WJ Bakker-Jonges LE Innate immunity to *Campylobacter jejuni* in Guillain–Barre syndrome. Ann Neurol (2015) 78(3):343–54. 10.1002/ana.24442 26017721

[96] Kabat AM Srinivasan N Maloy KJ. Modulation of immune development and function by intestinal microbiota. Trends Immunol (2014) 35(11):507–17. 10.1016/j.it.2014.07.010 25172617 PMC6485503

[97] Brodziak F Meharg C Blaut M Loh G. Differences in mucosal gene expression in the colon of two inbred mouse strains after colonization with commensal gut bacteria. PLoS One (2013) 8(8):e72317. 10.1371/journal.pone.0072317 23951309 PMC3739790

[98] Thompson CL Hofer MJ Campbell IL Holmes AJ. Community dynamics in the mouse gut microbiota: a possible role for IRF9-regulated genes in community homeostasis. PLoS One (2010) 5(4):e10335. 10.1371/journal.pone.0010335 20428250 PMC2859068

[99] Suprunenko T Hofer MJ. The emerging role of interferon regulatory factor 9 in the antiviral host response and beyond. Cytokine Growth Factor Rev (2016) 29:35–43. 10.1016/j.cytogfr.2016.03.002 26987614

[100] Sonnenburg JL Chen CT Gordon JI. Genomic and metabolic studies of the impact of probiotics on a model gut symbiont and host. PLoS Biol (2006) 4(12):e413. 10.1371/journal.pbio.0040413 17132046 PMC1661682

[101] Abt MC Osborne LC Monticelli LA Doering TA Alenghat T Sonnenberg GF Commensal bacteria calibrate the activation threshold of innate antiviral immunity. Immunity (2012) 37(1):158–70. 10.1016/j.immuni.2012.04.011 22705104 PMC3679670

[102] Ganal SC Sanos SL Kallfass C Oberle K Johner C Kirschning C Priming of natural killer cells by nonmucosal mononuclear phagocytes requires instructive signals from commensal microbiota. Immunity (2012) 37(1):171–86. 10.1016/j.immuni.2012.05.020 22749822

[103] McAleer JP Kolls JK. Maintaining poise: commensal microbiota calibrate interferon responses. Immunity (2012) 37(1):10–2. 10.1016/j.immuni.2012.07.001 22840839

[104] Contractor N Louten J Kim L Biron CA Kelsall BL Cutting edge: Peyer’s patch plasmacytoid dendritic cells (pDCs) produce low levels of type I interferons: possible role for IL-10, TGFbeta, and prostaglandin E2 in conditioning a unique mucosal pDC phenotype. J Immunol (2007) 179(5):2690–4. 10.4049/jimmunol.179.5.2690 17709480

[105] Tezuka H Abe Y Asano J Sato T Liu J Iwata M Prominent role for plasmacytoid dendritic cells in mucosal T cell-independent IgA induction. Immunity (2011) 34(2):247–57. 10.1016/j.immuni.2011.02.002 21333555

[106] Sun L Miyoshi H Origanti S Nice TJ Barger AC Manieri NA Type I interferons link viral infection to enhanced epithelial turnover and repair. Cell Host Microbe (2015) 17(1):85–97. 10.1016/j.chom.2014.11.004 25482432 PMC4297260

[107] Katlinskaya YV Katlinski KV Lasri A Li N Beiting DP Durham AC Type I interferons control proliferation and function of the intestinal epithelium. Mol Cell Biol (2016) 36(7):1124–35. 10.1128/MCB.00988-15 26811327 PMC4800802

[108] Liu J Carvalho LP Bhattacharya S Carbone CJ Kumar KG Leu NA Mammalian casein kinase 1alpha and its leishmanial ortholog regulate stability of IFNAR1 and type I interferon signaling. Mol Cell Biol (2009) 29(24):6401–12. 10.1128/MCB.00478-09 19805514 PMC2786868

[109] Powrie F Leach MW Mauze S Caddle LB Coffman RL. Phenotypically distinct subsets of CD4+ T cells induce or protect from chronic intestinal inflammation in C. B-17 scid mice. Int Immunol (1993) 5(11):1461–71. 10.1093/intimm/5.11.1461 7903159

[110] Mottet C Uhlig HH Powrie F. Cutting edge: cure of colitis by CD4+CD25+ regulatory T cells. J Immunol (2003) 170(8):3939–43. 10.4049/jimmunol.170.8.3939 12682220

[111] Rauch I Muller M Decker T. The regulation of inflammation by interferons and their STATs. JAKSTAT (2013) 2(1):e23820. 10.4161/jkst.23820 24058799 PMC3670275

[112] Hofmann C Dunger N Grunwald N Hammerling GJ Hoffmann P Scholmerich J T cell-dependent protective effects of CpG motifs of bacterial DNA in experimental colitis are mediated by CD11c+ dendritic cells. Gut (2010) 59(10):1347–54. 10.1136/gut.2009.193177 20732920

[113] Shiow LR Rosen DB Brdickova N Xu Y An J Lanier LL CD69 acts downstream of interferon-alpha/beta to inhibit S1P1 and lymphocyte egress from lymphoid organs. Nature (2006) 440(7083):540–4. 10.1038/nature04606 16525420

[114] Munakata K Yamamoto M Anjiki N Nishiyama M Imamura S Iizuka S Importance of the interferon-alpha system in murine large intestine indicated by microarray analysis of commensal bacteria-induced immunological changes. BMC Genomics (2008) 9:192. 10.1186/1471-2164-9-192 18439305 PMC2408602

[115] Clevers HC Bevins CL. Paneth cells: maestros of the small intestinal crypts. Annu Rev Physiol (2013) 75:289–311. 10.1146/annurev-physiol-030212-183744 23398152

[116] Brand S Beigel F Olszak T Zitzmann K Eichhorst ST Otte JM IL-28A and IL-29 mediate antiproliferative and antiviral signals in intestinal epithelial cells and murine CMV infection increases colonic IL-28A expression. Am J Physiol Gastrointest Liver Physiol (2005) 289(5):G960–8. 10.1152/ajpgi.00126.2005 16051921

[117] Maloy KJ Powrie F Intestinal homeostasis and its breakdown in inflammatory bowel disease. Nature (2011) 474(7351):298–306. 10.1038/nature10208 21677746

[118] Jostins L Ripke S Weersma RK Duerr RH McGovern DP Hui KY Host-microbe interactions have shaped the genetic architecture of inflammatory bowel disease. Nature (2012) 491(7422):119–24. 10.1038/nature11582 23128233 PMC3491803

[119] Khor B Gardet A Xavier RJ. Genetics and pathogenesis of inflammatory bowel disease. Nature (2011) 474(7351):307–17. 10.1038/nature10209 21677747 PMC3204665

[120] Giles EM Sanders TJ McCarthy NE Lung J Pathak M MacDonald TT Regulation of human intestinal T-cell responses by type 1 interferon-STAT1 signaling is disrupted in inflammatory bowel disease. Mucosal Immunol (2016) 10:184–93. 10.1038/mi.2016.44 27220814

[121] Axtell RC de Jong BA Boniface K van der Voort LF Bhat R De Sarno P T helper type 1 and 17 cells determine efficacy of interferon-beta in multiple sclerosis and experimental encephalomyelitis. Nat Med (2010) 16(4):406–12. 10.1038/nm.2110 20348925 PMC3042885

[122] Gasche C Reinisch W Vogelsang H Potzi R Markis E Micksche M Prospective evaluation of interferon-alpha in treatment of chronic active Crohn’s disease. Dig Dis Sci (1995) 40(4):800–4. 10.1007/BF02064982 7720472

[123] Madsen SM Schlichting P Davidsen B Nielsen OH Federspiel B Riis P An open-labeled, randomized study comparing systemic interferon-alpha-2A and prednisolone enemas in the treatment of left-sided ulcerative colitis. Am J Gastroenterol (2001) 96(6):1807–15. 10.1111/j.1572-0241.2001.03875.x 11419834

[124] Nikolaus S Rutgeerts P Fedorak R Steinhart AH Wild GE Theuer D Interferon beta-1a in ulcerative colitis: a placebo controlled, randomised, dose escalating study. Gut (2003) 52(9):1286–90. 10.1136/gut.52.9.1286 12912859 PMC1773804

[125] Tilg H Vogelsang H Ludwiczek O Lochs H Kaser A Colombel JF A randomised placebo controlled trial of pegylated interferon alpha in active ulcerative colitis. Gut (2003) 52(12):1728–33. 10.1136/gut.52.12.1728 14633951 PMC1773891

[126] Pena-Rossi C Schreiber S Golubovic G Mertz-Nielsen A Panes J Rachmilewitz D Clinical trial: a multicentre, randomized, double-blind, placebo-controlled, dose-finding, phase II study of subcutaneous interferon-beta-la in moderately active ulcerative colitis. Aliment Pharmacol Ther (2008) 28(6):758–67. 10.1111/j.1365-2036.2008.03778.x 19145731

[127] Pena-Rossi C Hanauer SB Tomasevic R Hunter JO Shafran I Graffner H. Interferon beta-1a for the maintenance of remission in patients with Crohn’s disease: results of a phase II dose-finding study. BMC Gastroenterol (2009) 9:22. 10.1186/1471-230X-9-22 19302707 PMC2674451

[128] Mannon PJ Hornung RL Yang Z Yi C Groden C Friend J Suppression of inflammation in ulcerative colitis by interferon-beta-1a is accompanied by inhibition of IL-13 production. Gut (2011) 60(4):449–55. 10.1136/gut.2010.226860 20971977 PMC3430969

[129] Wang Y MacDonald JK Benchimol EI Griffiths AM Steinhart AH Panaccione R Type I interferons for induction of remission in ulcerative colitis. Cochrane Database Syst Rev (2015) (9):CD006790 10.1002/14651858.CD006790.pub3 26368001 PMC9196197

[130] Meresse B Malamut G Cerf-Bensussan N. Celiac disease: an immunological jigsaw. Immunity (2012) 36(6):907–19. 10.1016/j.immuni.2012.06.006 22749351

[131] Kumar V Wijmenga C Withoff S. From genome-wide association studies to disease mechanisms: celiac disease as a model for autoimmune diseases. Semin Immunopathol (2012) 34(4):567–80. 10.1007/s00281-012-0312-1 22580835 PMC3410018

[132] Cammarota G Cuoco L Cianci R Pandolfi F Gasbarrini G. Onset of coeliac disease during treatment with interferon for chronic hepatitis C. Lancet (2000) 356(9240):1494–5. 10.1016/S0140-6736(00)02880-4 11081540

[133] Adinolfi LE Durante-Mangoni E Andreana A Interferon and ribavirin treatment for chronic hepatitis C may activate celiac disease. Am J Gastroenterol (2001) 96(2):607–8. 10.1111/j.1572-0241.2001.03574.x 11232725

[134] Bourliere M Oules V Perrier H Mengotti C Onset of coeliac disease and interferon treatment. Lancet (2001) 357(9258):803–4. 10.1016/S0140-6736(05)71230-7 11253998

[135] Monteleone G Pender SL Alstead E Hauer AC Lionetti P McKenzie C Role of interferon alpha in promoting T helper cell type 1 responses in the small intestine in coeliac disease. Gut (2001) 48(3):425–9. 10.1136/gut.48.3.425 11171837 PMC1760133

[136] Martins EV Jr Gaburri AK Celiac disease onset after pegylated interferon and ribavirin treatment of chronic hepatitis C. Arq Gastroenterol (2004) 41(2):132–3. 10.1590/S0004-28032004000200012 15543388

[137] Gombosova L Jarcuska P Benova B Benicky M Lazurova I. Celiac disease manifested during the treatment of chronic hepatitis C by pegylated alpha interferon and ribavirin. Bratisl Lek Listy (2011) 112(6):360–2. 21692415

[138] Durante-Mangoni E Iardino P Resse M Cesaro G Sica A Farzati B Silent celiac disease in chronic hepatitis C: impact of interferon treatment on the disease onset and clinical outcome. J Clin Gastroenterol (2004) 38(10):901–5. 10.1097/00004836-200411000-00014 15492610

[139] Gravina AG Federico A Masarone M Cuomo A Tuccillo C Loguercio C Coeliac disease and C virus-related chronic hepatitis: a non association. BMC Res Notes (2012) 5:533. 10.1186/1756-0500-5-533 23009068 PMC3544570

[140] Monteleone G Pender SL Wathen NC MacDonald TT Interferon-alpha drives T cell-mediated immunopathology in the intestine. Eur J Immunol (2001) 31(8):2247–55. 10.1002/1521-4141(200108)31:8<2247::AID-IMMU2247>3.0.CO;2-4 11477536

